# Dataset of long-term monitoring of ground-dwelling ants (Hymenoptera: Formicidae) in the influence areas of a hydroelectric power plant on the Madeira River in the Amazon Basin

**DOI:** 10.3897/BDJ.6.e24375

**Published:** 2018-04-02

**Authors:** Itanna O. Fernandes, Jorge L.P. de Souza

**Affiliations:** 1 Instituto Nacional de Pesquisas da Amazônia - INPA, Coordenação em Biodiversidade - CBio, Av: André Araújo, 2936. Petrópolis. 69067-375, Manaus, Brazil; 2 Programa de Pós-Graduação em Ciência e Tecnologia para Recursos Amazônicos, Instituto de Ciências Exatas e Tecnologia (ICET), Itacoatiara, Brazil

**Keywords:** Formicidae, biodiversity, species occurrence, standardized sampling protocol, tropical forest.

## Abstract

**Background:**

Biodiversity loss is accelerating rapidly in response to increasing human influence on the Earth’s natural ecosystems. One way to overcome this problem is by focusing on places of human interest and monitoring the changes and impacts on the biodiversity. This study was conducted at six sites within the influence area of the Santo Antônio Hydroelectric Power Plant in the margins of the Madeira River in Rondônia State. The sites cover a latitudinal gradient of approximately 100 km in the Brazilian Amazon Basin. The sampling design included six sampling modules with six plots (transects) each, totaling 30 sampling plots. The transects were distributed with 0 km, 0.5 km, 1 km, 2 km, 3 km and 4 km, measured perpendicularly from the river margin towards the interior of the forest. For sampling the ground-dwelling ants, the study used the ALL (ants of the leaf litter) protocol, which is standardized globally in the inventories of ant fauna. For the purpose of impact indicators, the first two campaigns (September 2011 to November 2011) were carried out in the pre-filling period, while campaigns 3 to 10 (February 2012 to November 2014) were carried out during and after the filling of the hydroelectric reservoir. A total of 253 events with a total of 9,165 occurrences were accounted during the monitoring. The ants were distributed in 10 subfamilies, 68 genera and 324 species/morphospecies. The impact on ant biodiversity during the periods before and after filling was measured by ecological indicators and by the presence and absence of some species/morphospecies. This is the first study, as far as we know, including taxonomic and ecological treatment to monitor the impact of a hydroelectric power plant on ant fauna.

**New information:**

Until recently, most studies conducted on hydroelectric plants, located in the Amazon Basin, were carried out after the implementation of dams in order to assess their impacts on the environment and biodiversity (Benchimol and Peres 2015, Latrubesse et al. 2017, Sá-Oliveira et al. 2015). Recent studies on dam impacts have begun to be conducted prior to dam implementation (e.g. Bobrowiec and Tavares 2017, Fraga et al. 2014, Moser et al. 2014), thus providing a better overview of the impact and a better assessment of its magnitude.

## Introduction

Biodiversity loss is accelerating rapidly in response to increasing human influence on the Earth’s natural ecosystems (Pimm et al. 1995, Vitousek 1997). Knowing the spatial and temporal organization of species in natural environments is essential for the understanding and conservation of biodiversity (Barton et al. 2013), as well as fostering land management decisions (Evans and Viengkham 2001). Large-scale, spatially structured sampling is a powerful tool to help land managers decide where to pursue conservation action most effectively (Turner et al. 1995). Even today, it is difficult to access accurate information on the spatial distribution of most organisms and their relationships with environmental variables at large scales, despite the availability of many methods for biodiversity planning and conservation (Barlow et al. 2010, Gibson et al. 2011, Margules et al. 2002). There are databases on species richness (Costello et al. 2013), but richness alone has limited use for conservation, because it does not give information on many endemic species or the complementarity of species compositions between regions (Groc et al. 2014, Lamoreux et al. 2005, Sarkar and Margules 2002). Furthermore, most assessments of species–habitat relationships can be compromised if the sampling design of surveys is not spatially clear (Gotelli et al. 2011).

Invertebrate populations can indicate longer-term general ecosystem change, such as restoration of mine sites or climate change (e.g., McGeoch 1998, Bisevac and Majer 1999, Parmesan et al. 1999, York 2000). However, despite recognition that monitoring invertebrates is an important endeavour, widely accepted by national and international funding agencies, monitoring efforts have rarely generated returns commensurate to their investment. All too frequently, insect monitoring lacks both specific goals and a framework detailing how results will be integrated into management decision-making.

One way to overcome these situations is by using good bioindicators taxa, as well as ants, considered particularly useful for monitoring for a number of reasons. Ants are one of the most successful groups of organisms on the planet (Hölldobler and Wilson 1990). To date, approximately 13,360 species of ants (antcat.org), all eusocial, have been described and hundreds of new species are described each year. Ant biologists estimate that the Formicidae family could include no fewer than 20,000 species (Hölldobler and Wilson 1990). All species of ants occupy a nest structure, either temporarily or permanently. These structures can be preexisting cavities or even made their own bodies (e.g. army ants) that do not involve much, if any, excavation or direct modification of the surrounding environments (Guénard 2013). They are abundant and ubiquitous in both intact habitat and disturbed areas (Andersen 1990 Majer 1983, Hoffmann et al. 2000), sampling is relatively easy without requiring enormous expertise (Greenslade and Greenslade 1984, Fisher 1999, Agosti and Alonso 2000, Alonso 2000), and ants have proven sensitive and rapid responders to environmental variables (Campbell and Tanton 1981, Majer 1983 Andersen 1990). Moreover, ants are important functionally at many different trophic levels (Alonso 2000), and they play critical ecological roles in soil turnover and structure (Humphreys 1981, Lobry de Bruyn and Conacher 1994), nutrient cycling (Levieux 1983, Lal 1988), plant protection, seed dispersal, and seed predation (Ashton 1979, Beattie 1985, Christian 2001). Together, these qualities suggest ants merit monitoring for their own sake, as they provide high information content about an ecologically and numerically dominant group (Underwood and Fisher 2006). Despite the increased availability of methods for conservation planning, adequate information about the spatial distribution of biodiversity in large regions, such as the Amazon Basin, remains sparse for most biological groups (Margules et al. 2002).

More than a hundred hydropower dams have already been built in the Amazon Basin and numerous proposals for further dam constructions are under consideration (Latrubesse et al. 2017). Recent scientific reviews have considered the environmental impacts of damming Amazonian rivers (Davidson et al. 2012, Castello and Macedo 2015, Winemiller et al. 2016, Fearnside 2016). The accumulated negative environmental effects of existing dams, not to mention proposed dams (if constructed), have triggered massive hydrophysical and biotic disturbances affecting the Amazon Basin’s floodplains, estuaries and sediment plumes (Latrubesse et al. 2017), as well as causing losses in river connectivity (Anderson et al. 2018).

The Santo Antônio Hydroelectric Power Plant became operational at the beginning of 2016 in the Madeira River in Rondônia State. Prior to the construction of the Santo Antônio Plant, the fauna and flora of the impacted area were surveyed in environmental impact studies commissioned by the Brazilian Institute of Environment (IBAMA). The Santo Antônio Hydroelectric Power Plant and its accompanying reservoir represent the first time in history, as far as we know, in which a monitoring program of invertebrates was conducted to evaluate the influence before and after the total filling of the dam in the Amazon Basin.

## Project description

### Title

Environmental monitoring of ants (Hymenoptera: Formicidae) in the influence areas of the Santo Antônio Hydroelectric Power Plant in the Madeira River in the Brazilian Amazon

### Personnel

Itanna Oliveira Fernandes, Jorge Luiz Pereira de Souza

### Study area description

The study was conducted at six sites associated with the Brazilian Biodiversity Research Program (PPBio) — Pedras, Búfalos, Morrinhos, Jaci-Paraná MD, Jaci-Paraná ME and Teotônio modules — within the influence area of the Santo Antônio Hydroelectric Power Plant in the margins of the Madeira River in Rondônia State.

### Design description

Ants were sampled in permanent plots with five samples per sampling method. We used the RAPELD sampling design, which is based on a system of trails and permanent plots where a diverse range of taxa can be sampled (Costa and Magnusson 2010, Magnusson et al. 2005, Magnusson et al. 2013). The permanent plots are 250 m long and positioned to follow terrain contours to minimize the effects of topographical variation within plots. In each module, transects have a 1 km distance from each other, following the same spatial design.

## Sampling methods

### Study extent

The sites cover a latitudinal gradient of approximately 100 km in the Brazilian Amazon Basin. The sampling design included six sampling modules with six transects (Pedras, Búfalos, Morrinhos, Jaci-Paraná MD, Jaci-Paraná ME and Teotônio modules), each totalling 30 sampling plots. The transects were distributed 0 km, 0.5 km, 1 km, 2 km, 3 km and 4 km from the river's edge, measured perpendicularly from the river margin towards the interior of the forest. For the purpose of impact indicators, the first two campaigns (September 2011 to November 2011) were carried out in the pre-filling period, while campaigns 3 to 10 (February 2012 to November 2014) were carried out after the filling of the hydroelectric reservoir. The campaigns were conducted during the dry and rainy seasons of the Amazon over four years, with intervals of three months between each campaign (whenever possible).

### Sampling description

**Figure 1.**
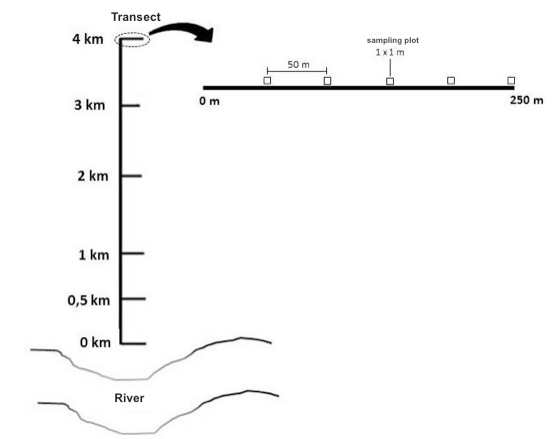
Transects of each module to collect ants in the influence areas of the Santo Antônio Hydroelectric Power Plant, Porto Velho - RO, with perpendicular distances from the river margin. In details are each transect with a 1 km distance from each other following the same spatial design and each sampling plot in the permanent plots of 250 m length.

Ants were sampled in permanent plots with five samples per sampling method along the transects 0 km, 0.5 km, 1 km, 2 km, 3 km and 4 km (Fig. 1). We used the RAPELD sampling design, which is based on a system of trails and permanent plots where a diverse range of taxa can be sampled (Costa and Magnusson 2010, Magnusson et al. 2005, Magnusson et al. 2013). The permanent plots are 250 m long and positioned to follow terrain contours to minimize the effects of topographical variation within plots. In each site, plots were 1 km apart from each other, following the same spatial design.

**Figure 2.**
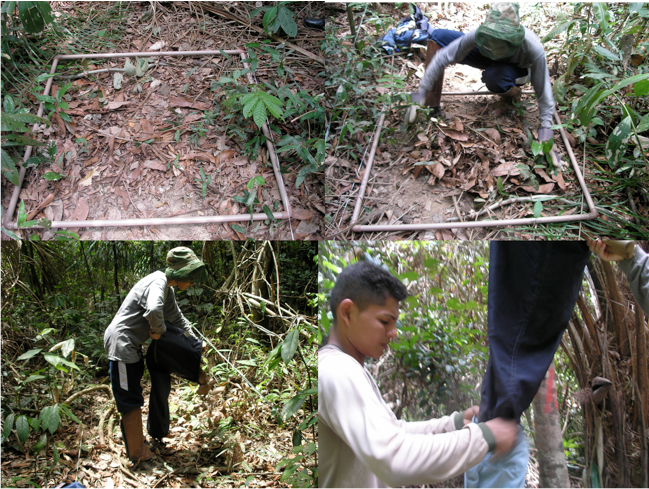
Sample from 1 m^2^ leaf litter of each sampling plot located at 50 m intervals along the transect and mesh sieve used to separate the leaves from the invertebrates.

**Figure 3.**
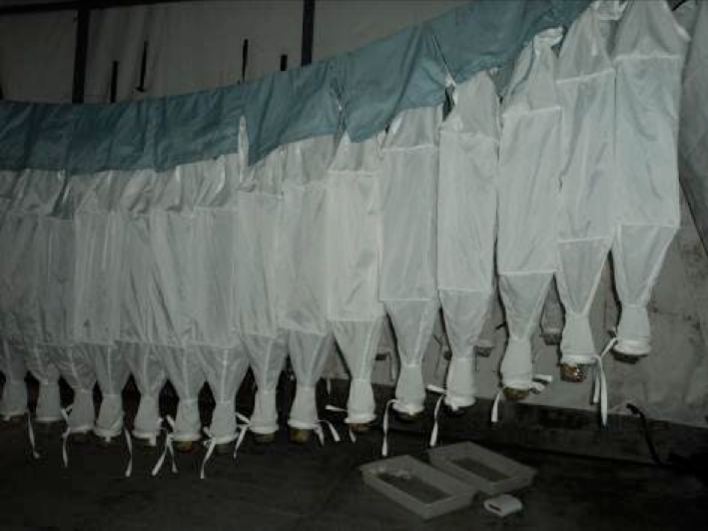
Mini-Winkler extractors composed by a mesh bag filled with sifted sample inside and a cotton bag outside. In response to the drying, the ants migrate from the suspended sample and fall into a container partially filled with alcohol at the bottom of the bag.

The protocol adopted for collection of litter ants is called the ALL protocol (leaflet ants), which is globally standardized on inventories of a litter of ant fauna (Agosti and Alonso 2000). Ground-dwelling ants collected in plots using litter samples were processed in Winkler extractors. Litter-dwelling ants were sampled from a 1 m^2^ litter in sampling plots located at 50 m intervals along the center line of each transect. Using a Winkler extractor with a 1 cm^2^ mesh sieve, the leaves were sifted through a wire sieve of 1 cm^2^ mesh size by shaking the sifter vigorously at least 15 times. The ants were extracted from the sifted litter and placed in a mesh bag inside a cotton bag for 24 hours (Fig. 2). If the sifted leaf litter volume exceeded the capacity of a single mini-Winkler extractor, a second extractor was used. In behavioural response to litter drying, the ants migrate from the suspended sample and fall into a container partially filled with alcohol at the bottom of the bag (Agosti et al. 2000, Bestelmeyer et al. 2000) (Fig. 3). The litter-sampling procedures were undertaken between 8:00 am and 5:00 pm. All ants were first identified to genus using the taxonomic keys provided by Baccaro et al. 2015. Then, they were sorted into species and morphospecies. We used available taxonomic keys or compared with specimens in collections previously identified by experts. A unique identification was given for each morphospecies based on morphological differences from related species. The morphotyping was the same for all collection sites. Vouchers are deposited in the invertebrate collection of the National Institute of Amazonian Research (INPA).

## Geographic coverage

### Description

Areas of Santo Antônio Hydroelectric Power-Plant in Rondônia, Brazil.

### Coordinates

-9.25 and -8.59 Latitude; -64.45 and -63.88 Longitude.

## Taxonomic coverage

### Description

The ants were identified by species and morphospecies, as well as subfamily. Some genera were recorded for the first time in South America (*Syscia* Roger, 1861) and others in Rondônia State (*Nylanderia* Emery, 1906; *Eurhopalothrix* Brown & Kempf, 1961; *Lachnomyrmex* Wheeler, 1910; *Mycetarotes* Emery, 1913; *Mycetophylax* Emery, 1913; *Nesomyrmex* Wheeler, 1910; and *Rhopalothrix* Mayr, 1870). We also obtained new records of the following species for Rondônia State: *Fulakora degenerata*, *Tapinoma melanocephalum*, *Neivamyrmex adnepos*, *Gnamptogenys acuminata*, *Gnamptogenys caelata*, *Gnamptogenys kempfi*, *Cephalotes pellans*, *Hylomyrma immanis*, *Rogeria blanda*, *Strumigenys deinomastax*, *Strumigenys infidelis*, *Wasmannia rochai*, *Wasmannia scrobifera*, *Anochetus mayri*, *Anochetus neglectus*, *Anochetus targionii* and *Leptogenys unistimulosa*. A total of 46,342 individuals were collected during four years of field collections. A list of all the ants identified in subfamilies (10), genera (68) and species/morphospecies (324). More information about the ecological data and occurence is available in Suppl. materials 1, 2

### Taxa included

**Table:** 

Rank	Scientific Name	Common Name
family	Formicidae Latreille, 1809	ant
subfamily	Agroecomyrmecinae Carpenter, 1930	ant
genus	*Tatuidris* Brown & Kempf, 1968	ant
species	*Tatuidris tatusia* Brown & Kempf, 1968	ant
subfamily	Amblyoponinae Forel, 1893	ant
genus	*Fulakora* Mann, 1919	ant
species	*Fulakora degenerata* (Borgmeier, 1957)	ant
genus	*Prionopelta* Mayr, 1866	ant
species	*Prionopelta* sp. 1	ant
subfamily	Dolichoderinae Forel, 1878	ant
genus	*Azteca* Forel, 1878	ant
species	Azteca cf. chartiffex Emery, 1896	ant
species	*Azteca* sp. 1	ant
species	*Azteca* sp. 2	ant
species	*Azteca* sp. 3	ant
species	*Azteca* sp. 4	ant
species	*Azteca* sp. 5	ant
genus	*Dolichoderus* Lund, 1831	ant
species	*Dolichoderus bidens* (Linnaeus, 1758)	ant
species	*Dolichoderus bispinosus* (Olivier, 1792)	ant
species	*Dolichoderus cogitans* Forel, 1912	ant
species	*Dolichoderus debilis* Emery, 1890	ant
species	*Dolichoderus decollatus* Smith, 1858	ant
species	*Dolichoderus imitator* Emery, 1894	ant
species	*Dolichoderus longicollis* MacKay, 1993	ant
species	*Dolichoderus septemspinosus* Emery, 1894	ant
species	*Dolichoderus* sp. 1	ant
genus	*Linepithema* Mayr, 1866	ant
species	*Linepithema* sp. 1	ant
genus	*Tapinoma* Foerster, 1850	ant
species	*Tapinoma melanocephalum* (Fabricius, 1793)	ant
species	*Tapinoma* sp. 1	ant
subfamily	Dorylinae Leach, 1815	ant
genus	*Cheliomyrmex* Mayr, 1870	ant
species	*Cheliomyrmex megalonyx* Wheeler, 1921	ant
genus	*Eciton* Latreille, 1804	ant
species	*Eciton burchellii* (Westwood, 1842)	ant
genus	*Labidus* Jurine, 1807	ant
species	*Labidus praedator* (Smith, 1858)	ant
species	*Labidus spininodis* (Emery, 1890)	ant
genus	*Neivamyrmex* Borgmeier, 1940	ant
species	*Neivamyrmex adnepos* (Wheeler, 1922)	ant
species	*Neivamyrmex angustinodis* (Emery, 1888)	ant
species	*Neivamyrmex* sp. 3	ant
genus	*Neocerapachys* Borowiec, 2016	ant
species	*Neocerapachys splendens* (Borgmeier, 1957)	ant
genus	*Syscia* Roger, 1861	ant
species	*Syscia augustae* (Wheeler, 1902)	ant
subfamily	Ectatomminae Emery, 1895	ant
genus	*Ectatomma* Smith, 1858	ant
species	*Ectatomma brunneum* Smith, 1858	ant
species	*Ectatomma edentatum* Roger, 1863	ant
species	*Ectatomma lugens* Emery, 1894	ant
genus	*Gnamptogenys* Roger, 1863	ant
species	*Gnamptogenys acuminata* (Emery, 1896)	ant
species	*Gnamptogenys caelata* Kempf, 1967	ant
species	*Gnamptogenys ericae* (Forel, 1912)	ant
species	*Gnamptogenys haenschi* (Emery, 1902)	ant
species	*Gnamptogenys horni* (Santschi, 1929)	ant
species	*Gnamptogenys kempfi* Lenko, 1964	ant
species	*Gnamptogenys moelleri* (Forel, 1912)	ant
species	*Gnamptogenys pleurodon* (Emery, 1896)	ant
species	*Gnamptogenys relicta* (Mann, 1916)	ant
species	*Gnamptogenys* sp. 1	ant
species	*Gnamptogenys* sp. 11	ant
species	*Gnamptogenys* sp. 3	ant
species	*Gnamptogenys* sp. 5	ant
species	*Gnamptogenys tortuolosa* (Smith, 1858)	ant
genus	*Typhlomyrmex* Mayr, 1862	ant
species	*Typhlomyrmex* sp. 1	ant
subfamily	Formicinae Latreille, 1809	ant
genus	*Acropyga* Roger, 1862	ant
species	*Acropyga* sp. 1	ant
genus	*Brachymyrmex* Mayr, 1868	ant
species	*Brachymyrmex* sp. 1	ant
species	*Brachymyrmex* sp. 2	ant
species	*Brachymyrmex* sp. 3	ant
species	*Brachymyrmex* sp. 4	ant
species	*Brachymyrmex* sp. 5	ant
species	*Brachymyrmex* sp. 6	ant
genus	*Camponotus* Mayr, 1861.	ant
species	*Camponotus atriceps* (Smith, 1858)	ant
species	*Camponotus blandus* (Smith, 1858)	ant
species	*Camponotus cameranoi* Emery, 1894	ant
species	*Camponotus crassus* Mayr, 1862	ant
species	*Camponotus fastigatus* Roger, 1863	ant
species	*Camponotus femoratus* (Fabricius, 1804)	ant
species	*Camponotus novogranadensis* Mayr, 1870	ant
species	*Camponotus rapax* (Fabricius, 1804)	ant
species	*Camponotus rectangularis* Emery, 1890	ant
species	*Camponotus sericeiventris* (Guérin-Méneville, 1838)	ant
species	*Camponotus* sp. 5	ant
species	*Camponotus* sp. 6	ant
genus	*Gigantiops* Roger, 1863	ant
species	*Gigantiops destructor* (Fabricius, 1804)	ant
genus	*Nylanderia* Emery, 1906	ant
species	Nylanderia cf. caeciliae (Forel, 1899)	ant
species	Nylanderia cf. fulva (Mayr, 1862)	ant
species	Nylanderia cf. guatemalensis (Forel, 1885)	ant
species	*Nylanderia* sp. 3	ant
species	*Nylanderia* sp. 5	ant
subfamily	Myrmicinae Lepeletier de Saint-Fargeau, 1835	ant
genus	*Acromyrmex* Mayr, 1865	ant
species	Acromyrmex cf. subterraneus (Forel, 1893)	ant
genus	*Allomerus* Mayr, 1878	ant
species	*Allomerus octoarticulatus* Mayr, 1878	ant
genus	*Apterostigma* Mayr, 1865	ant
species	*Apterostigma auriculatum* Wheeler, 1925	ant
species	Apterostigma gr. pilosum	ant
genus	*Atta* Fabricius, 1804	ant
species	*Atta cephalotes* (Linnaeus, 1758)	ant
species	*Atta sexdens* (Linnaeus, 1758)	ant
genus	*Basiceros* Schulz, 1906	ant
species	*Basiceros militaris* (Weber, 1950)	ant
genus	*Blepharidatta* Wheeler, 1915	ant
species	*Blepharidatta brasiliensis* Wheeler, 1915	ant
genus	*Carebara* Westwood, 1840	ant
species	Carebara gr. lignata	ant
species	*Carebara* sp. 1	ant
species	*Carebara* sp. 2	ant
species	*Carebara* sp. 5	ant
species	*Carebara urichi* (Wheeler, 1922)	ant
genus	*Cephalotes* Latreille, 1802	ant
species	*Cephalotes atratus* (Linnaeus, 1758)	ant
species	*Cephalotes minutus* (Fabricius, 1804)	ant
species	*Cephalotes pellans* De Andrade, 1999	ant
species	*Cephalotes pusillus* (Klug, 1824)	ant
species	*Cephalotes* sp. 1	ant
species	*Cephalotes* sp. 2	ant
species	*Cephalotes* sp. 3	ant
genus	*Crematogaster* Lund, 1831	ant
species	*Crematogaster acuta* (Fabricius, 1804)	ant
species	*Crematogaster brasiliensis* Mayr, 1878	ant
species	*Crematogaster carinata* Mayr, 1862	ant
species	*Crematogaster curvispinos*a Mayr, 1862	ant
species	*Crematogaster flavosensitiva* Longino, 2003	ant
species	*Crematogaster limata* Smith, 1858	ant
species	*Crematogaster longispina* Emery, 1890	ant
species	*Crematogaster nigropilosa* Mayr, 1870	ant
species	*Crematogaster sotobosque* Longino, 2003	ant
species	*Crematogaster* sp. 2	ant
species	*Crematogaster stollii* Forel, 1885	ant
species	*Crematogaster tenuicula* Forel, 1904	ant
genus	*Cyphomyrmex* Mayr, 1862	ant
species	*Cyphomyrmex laevigatus* Weber, 1938	ant
species	*Cyphomyrmex minutus* Mayr, 1862	ant
species	*Cyphomyrmex peltatus* Kempf, 1966	ant
species	*Cyphomyrmex rimosus* (Spinola, 1851)	ant
species	Cyphomyrmex cf. salvini Forel, 1899	ant
species	*Cyphomyrmex* sp. 12	ant
species	*Cyphomyrmex* sp. 13	ant
species	*Cyphomyrmex* sp. 3	ant
species	*Cyphomyrmex* sp. 4	ant
genus	*Eurhopalothrix* Brown & Kempf, 1961	ant
species	*Eurhopalothrix pilulifera* Brown & Kempf, 1960	ant
genus	*Hylomyrma* Forel, 1912	ant
species	*Hylomyrma dentiloba* (Santschi, 1931)	ant
species	Hylomyrma cf. dolichops Kempf, 1973	ant
species	*Hylomyrma immanis* Kempf, 1973	ant
species	*Hylomyrma longiscapa* Kempf, 1961	ant
species	Hylomyrma cf. reitteri (Mayr, 1887)	ant
species	*Hylomyrma* sp. 2	ant
species	*Hylomyrma* sp. 3	ant
genus	*Lachnomyrmex* Wheeler, 1910	ant
species	*Lachnomyrmex* sp. 1	ant
genus	*Megalomyrmex* Forel, 1885	ant
species	*Megalomyrmex balzani* Emery, 1894	ant
species	*Megalomyrmex cuatiara* Brandão, 1990	ant
species	*Megalomyrmex drifti* Kempf, 1961	ant
species	*Megalomyrmex goeldii* Forel, 1912	ant
species	*Megalomyrmex leoninus* Forel, 1885	ant
species	*Megalomyrmex* sp. 2	ant
species	*Megalomyrmex* sp. 5	ant
species	*Megalomyrmex* sp. 8	ant
species	*Megalomyrmex wallacei* Mann, 1916	ant
genus	*Monomorium* Mayr, 1855	ant
species	*Monomorium pharaonis* (Linnaeus, 1758)	ant
genus	*Mycetarotes* Emery, 1913	ant
species	*Mycetarotes* sp. 1	ant
genus	*Mycetophylax* Emery, 1913	ant
species	Mycetophylax cf. lectus (Forel, 1911)	ant
species	*Mycetophylax strigatus* (Mayr, 1887)	ant
genus	*Mycocepurus* Forel, 1893	ant
species	*Mycocepurus goeldii* (Forel, 1893)	ant
species	*Mycocepurus* sp. 1	ant
species	*Mycocepurus* sp. 2	ant
species	*Mycocepurus* sp. 3	ant
genus	*Myrmicocrypta* Smith, 1860	ant
species	*Myrmicocrypta* sp. 1	ant
species	*Myrmicocrypta* sp. 2	ant
genus	*Nesomyrmex* Wheeler, 1910	ant
species	*Nesomyrmex pleuriticus* (Kempf, 1959)	ant
genus	*Ochetomyrmex* Mayr, 1878	ant
species	*Ochetomyrmex semipolitus* Mayr, 1878	ant
genus	*Octostruma* Forel, 1912	ant
species	*Octostruma balzani* (Emery, 1894)	ant
species	*Octostruma iheringi* (Emery, 1888)	ant
species	*Octostruma* sp. 1	ant
species	*Octostruma* sp. 2	ant
species	*Octostruma* sp. 3	ant
genus	*Oxyepoecus* Santschi, 1926	ant
species	*Oxyepoecus ephippiatus* Albuquerque & Brandão, 2004	ant
genus	*Pheidole* Westwood, 1839	ant
species	*Pheidole fracticeps* Wilson, 2003	ant
species	*Pheidole biconstricta* Mayr, 1870	ant
species	*Pheidole flavens* Roger, 1863	ant
species	*Pheidole vorax* (Fabricius, 1804)	ant
species	*Pheidole* sp. 1	ant
species	*Pheidole* sp. 4	ant
species	*Pheidole* sp. 6	ant
species	*Pheidole* sp. 4	ant
species	*Pheidole* sp. 6	ant
species	*Pheidole* sp. 10	ant
species	*Pheidole* sp. 11	ant
species	*Pheidole* sp. 12	ant
species	*Pheidole* sp. 14	ant
species	*Pheidole* sp. 15	ant
species	*Pheidole* sp. 16	ant
species	*Pheidole* sp. 17	ant
species	*Pheidole* sp. 18	ant
species	*Pheidole* sp. 19	ant
species	*Pheidole* sp. 2	ant
species	*Pheidole* sp. 20	ant
species	*Pheidole* sp. 21	ant
species	*Pheidole* sp. 22	ant
species	*Pheidole* sp. 23	ant
species	*Pheidole* sp. 24	ant
species	*Pheidole* sp. 26	ant
species	*Pheidole* sp. 27	ant
species	*Pheidole* sp. 28	ant
species	*Pheidole* sp. 29	ant
species	*Pheidole* sp. 3	ant
species	*Pheidole* sp. 30	ant
species	*Pheidole* sp. 32	ant
species	*Pheidole* sp. 40	ant
species	*Pheidole* sp. 41	ant
species	*Pheidole* sp. 42	ant
species	*Pheidole* sp. 43	ant
species	*Pheidole* sp. 44	ant
species	*Pheidole* sp. 45	ant
species	*Pheidole* sp. 46	ant
species	*Pheidole* sp. 47	ant
species	*Pheidole* sp. 48	ant
species	*Pheidole* sp. 49	ant
species	*Pheidole* sp. 5	ant
species	*Pheidole* sp. 50	ant
species	*Pheidole* sp. 51	ant
species	*Pheidole* sp. 52	ant
species	*Pheidole* sp. 53	ant
species	*Pheidole* sp. 54	ant
species	*Pheidole* sp. 55	ant
species	*Pheidole* sp. 7	ant
species	*Pheidole* sp. 8	ant
species	*Pheidole* sp. 9	ant
genus	*Rhopalothrix* Mayr, 1870	ant
species	*Rhopalothrix* sp. 1	ant
species	*Rhopalothrix* sp. 2	ant
genus	*Rogeria* Emery, 1894	ant
species	*Rogeria alzatei* Kugler, 1994	ant
species	Rogeria cf. belti Mann, 1922	ant
species	*Rogeria blanda* (Smith, 1858)	ant
species	Rogeria cf. cornuta Kugler, 1994	ant
species	Rogeria cf. cuneola Kugler, 1994	ant
species	*Rogeria leptonana* Kugler, 1994	ant
species	*Rogeria* sp. 1	ant
species	*Rogeria* sp. 2	ant
genus	*Sericomyrmex* Mayr, 1865	ant
species	*Sericomyrmex* sp. 1	ant
species	*Sericomyrmex* sp. 2	ant
genus	*Solenopsis* Westwood, 1840	ant
species	Solenopsis cf. castor Forel, 1893	ant
species	Solenopsis cf. clytemnestra Emery, 1896	ant
species	*Solenopsis geminata* (Fabricius, 1804)	ant
species	Solenopsis gr. molesta	ant
species	Solenopsis cf. loretana Santschi, 1936	ant
species	Solenopsis cf. saevissima (Smith, 1855)	ant
species	*Solenopsis* sp. 3	ant
species	*Solenopsis* sp. 5	ant
species	*Solenopsis* sp. 7	ant
species	*Solenopsis substituta* Santschi, 1925	ant
genus	*Stegomyrmex* Emery, 1912	ant
species	Stegomyrmex cf. olindae Feitosa, Brandão & Diniz, 2008	ant
genus	*Strumigenys* Smith, 1860	ant
species	*Strumigenys appretiata* (Borgmeier, 1954)	ant
species	*Strumigenys beebei* (Wheeler, 1915)	ant
species	*Strumigenys deinomastax* (Bolton, 2000)	ant
species	*Strumigenys denticulata* Mayr, 1887	ant
species	*Strumigenys elongata* Roger, 1863	ant
species	*Strumigenys infidelis* Santschi, 1919	ant
species	*Strumigenys inusitata* (Lattke, 1992)	ant
species	Strumigenys cf. perparva Brown, 1958	ant
species	*Strumigenys smithii* Forel, 1886	ant
species	*Strumigenys* sp. 1	ant
species	*Strumigenys* sp. 10	ant
species	*Strumigenys* sp. 13	ant
species	*Strumigenys* sp. 14	ant
species	*Strumigenys* sp. 15	ant
species	*Strumigenys* sp. 2	ant
species	*Strumigenys* sp. 3	ant
species	*Strumigenys* sp. 4	ant
species	*Strumigenys* sp. 5	ant
species	*Strumigenys* sp. 6	ant
species	*Strumigenys* sp. 7	ant
species	*Strumigenys* sp. 8	ant
species	*Strumigenys* sp. 9	ant
species	Strumigenys cf. trinidadensis Wheeler, 1922	ant
species	*Strumigenys trudifera* Kempf & Brown, 1969	ant
species	*Strumigenys zeteki* (Brown, 1959)	ant
genus	*Trachymyrmex* Forel, 1893	ant
species	Trachymyrmex cf. bugnioni (Forel, 1912)	ant
species	Trachymyrmex cf. cornetzi (Forel, 1912)	ant
species	Trachymyrmex cf. diversus Mann, 1916	ant
species	Trachymyrmex cf. farinosus (Emery, 1894)	ant
species	Trachymyrmex cf. mandibularis Weber, 1938	ant
species	Trachymyrmex cf. opulentus (Mann, 1922)	ant
species	Trachymyrmex cf. ruthae Weber, 1937	ant
species	*Trachymyrmex* sp. 10	ant
species	*Trachymyrmex* sp. 3	ant
species	*Trachymyrmex* sp. 7	ant
species	*Trachymyrmex* sp. 8	ant
species	*Trachymyrmex* sp. 9	ant
genus	*Tranopelta* Mayr, 1866	ant
species	*Tranopelta gilva* Mayr, 1866	ant
species	*Tranopelta* sp. 1	ant
genus	*Wasmannia* Forel, 1893	ant
species	*Wasmannia auropunctata* (Roger, 1863)	ant
species	*Wasmannia rochai* Forel, 1912	ant
species	*Wasmannia scrobifera* Kempf, 1961	ant
species	*Wasmannia* sp. 1	ant
subfamily	Ponerinae Lepeletier de Saint-Fargeau, 1835	ant
genus	*Anochetus* Mayr, 1861	ant
species	*Anochetus diegensis* Forel, 1912	ant
species	*Anochetus emarginatus* (Fabricius, 1804)	ant
species	*Anochetus horridus* Kempf, 1964	ant
species	*Anochetus mayri* Emery, 1884	ant
species	*Anochetus neglectus* Emery, 1894	ant
species	*Anochetus targionii* Emery, 1894	ant
genus	*Dinoponera* Roger, 1861	ant
species	*Dinoponera gigantea* (Perty, 1833)	ant
genus	*Hypoponera* Santschi, 1938	ant
species	*Hypoponera* sp. 1	ant
species	*Hypoponera* sp. 16	ant
species	*Hypoponera* sp. 2	ant
species	*Hypoponera* sp. 3	ant
species	*Hypoponera* sp. 4	ant
species	*Hypoponera* sp. 5	ant
species	*Hypoponera* sp. 6	ant
species	*Hypoponera* sp. 7	ant
species	*Hypoponera* sp. 8	ant
species	*Hypoponera* sp. 9	ant
genus	*Leptogenys* Roger, 1861	ant
species	*Leptogenys unistimulosa* Roger, 1863	ant
genus	*Mayaponera* Schmidt & Shattuck, 2014	ant
species	*Mayaponera constricta* (Mayr, 1884)	ant
genus	*Neoponera* Emery, 1901	ant
species	*Neoponera apicalis* (Latreille, 1802)	ant
species	*Neoponera cavinodis* Mann, 1916	ant
species	*Neoponera commutata* (Roger, 1860)	ant
species	*Neoponera laevigata* (Smith, 1858)	ant
species	*Neoponera unidentata* (Mayr, 1862)	ant
species	*Neoponera venusta* Forel, 1912	ant
species	*Neoponera verenae* Forel, 1922	ant
genus	*Odontomachus* Latreille, 1804	ant
species	*Odontomachus bauri* Emery, 1892	ant
species	*Odontomachus caelatus* Brown, 1976	ant
species	*Odontomachus chelifer* (Latreille, 1802)	ant
species	*Odontomachus haematodus* (Linnaeus, 1758)	ant
species	*Odontomachus hastatus* (Fabricius, 1804)	ant
species	*Odontomachus laticeps* Roger, 1861	ant
species	*Odontomachus meinerti* Forel, 1905	ant
species	*Odontomachus* sp. 1	ant
species	*Odontomachus* sp. 2	ant
genus	*Pachycondyla* Smith, 1858	ant
species	*Pachycondyla crassinoda* (Latreille, 1802)	ant
species	*Pachycondyla harpax* (Fabricius, 1804)	ant
species	*Pachycondyla impressa* (Roger, 1861)	ant
species	*Pachycondyla* sp. 1	ant
species	*Pachycondyla* sp. 2	ant
species	*Pachycondyla* sp. 3	ant
species	*Pachycondyla striata* Smith, 1858	ant
genus	*Pseudoponera* Emery, 1900	ant
species	*Pseudoponera stigma* (Fabricius, 1804)	ant
genus	*Rasopone* Schmidt & Shattuck, 2014	ant
species	*Rasopone arhuaca* (Forel, 1901)	ant
genus	*Simopelta* Mann, 1922	ant
species	*Simopelta anomma* Fernandes et al., 2015	ant
species	*Simopelta jeckylli* (Mann, 1916)	ant
genus	*Thaumatomyrmex* Mayr, 1887	ant
species	*Thaumatomyrmex atrox* Weber, 1939	ant
subfamily	Proceratiinae Emery, 1895	ant
genus	*Discothyrea* Roger, 1863	ant
species	*Discothyrea denticulata* Weber, 1939	ant
species	*Discothyrea humilis* Weber, 1939	ant
species	*Discothyrea sexarticulata* Borgmeier, 1954	ant
subfamily	Pseudomyrmecinae Smith, 1952	ant
genus	*Pseudomyrmex* Lund, 1831	ant
species	*Pseudomyrmex ita* (Forel, 1906)	ant
species	*Pseudomyrmex simplex* (Smith, 1877)	ant
species	*Pseudomyrmex* sp. 2	ant
species	*Pseudomyrmex* sp. 3	ant
species	*Pseudomyrmex tenuis* (Fabricius, 1804)	ant
species	*Pseudomyrmex termitarius* (Smith, 1855)	ant

## Traits coverage

### Data coverage of traits

PLEASE FILL IN TRAIT INFORMATION HERE

## Temporal coverage

### Notes

2011-09-02 through 2011-09-09, 2011-11-17 through 2012-12-03, 2012-02-28 through 2012-03-12, 2012-05-30 through 2012-06-11, 2013-09-19 through 2013-01-31, 2013-04-18 through 2013-04-28, 2013-06-28 through 2013-07-05, 2013-10-20 through 2013-09-26, 2014-01-17 through 2014-01-27, 2014-11-13 through 2014-11-23

## Collection data

### Collection name

Instituto Nacional de Pesquisas da Amazônia - INPA/ Coleção de Invertebrados/ HYM

### Specimen preservation method

alcohol, pinned

## Usage rights

### Use license

Other

### IP rights notes

This work is licensed under a Creative Commons Attribution Non Commercial (CC-BY-NC) 4.0 License.

## Data resources

### Data package title

Environmental monitoring of ants (Hymenoptera: Formicidae) in the influence areas of Santo Antônio Hydroelectric Power-Plant in Rondônia, Brazil.

### Alternative identifiers

914c3b86-f2a1-4d5e-b343-b2597b9d4542, https://ipt.sibbr.gov.br/sibbr/resource?r=ant_monitoring_in_santo_antonio_hydroelectric_power_plant_rondonia

### Number of data sets

2

### Data set 1.

#### Data set name

Environmental monitoring of ants (Hymenoptera: Formicidae) in the influence areas of Santo Antônio Hydroelectric Power-Plant in Rondônia, Brazil.

#### Data format

Darwin Core

#### Number of columns

26

#### Character set

Event

#### Description

**Figure 4.**
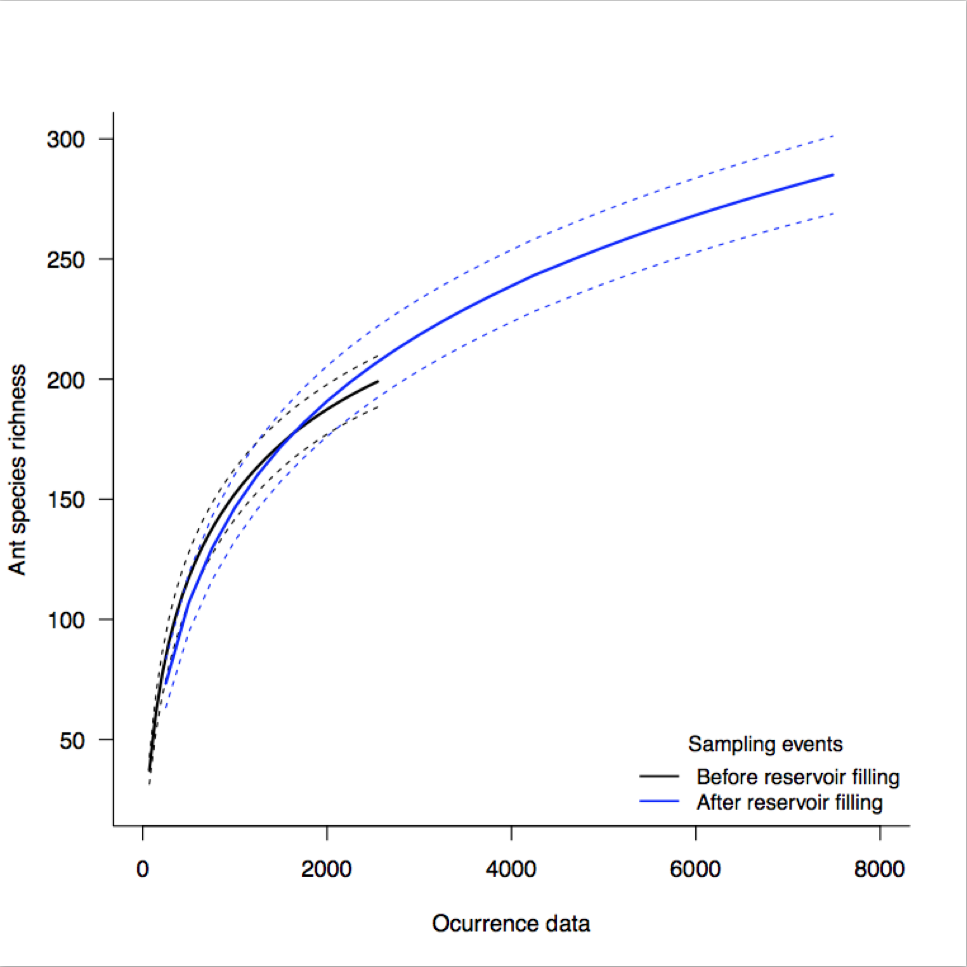
Species occurrence before and after reservoir filling in the Santo Antônio Hydroelectric Power Plant. Dotted lines mark the 95% confidence intervals.

Biodiversity loss is accelerating rapidly in response to increasing human influence on the Earth’s natural ecosystems. One way to overcome this problem is by focusing on places of human interest and monitoring the changes and impacts on the biodiversity. This study was conducted at six sites within the influence area of the Santo Antônio Hydroelectric Power Plant in the margins of the Madeira River, Rondônia. The sites cover a latitudinal gradient of approximately 100 km in the Brazilian Amazon Basin. The sampling design included six sampling modules with six transects in each module, totaling 30 sampling plots in each module. Transects were distrubuted with 0 km, 0.5 km, 1 km, 2 km, 3 km, and 4 km, measured perpendicularly from the river margin towards the interior of the forest. For sampling the ground-dwelling ants, we used the ALL (ants of the leaf litter) protocol, which is standardized globally in the inventories of ant fauna. For the purpose of impact indicators, the first two campaigns (September 2011 to November 2011) were carried out in the pre-filling period, while campaigns 3 to 10 (Febuary 2012 to November 2014) were carried out during and after the filling of the hydroelectric reservoir. A total of 253 events with a total of 9.165 occurrences were accounted during the monitoring. The ants were distributed in 10 subfamilies, 68 genera, and 324 species/morphospecies (Fig. 4). The impact on ant biodiversity during the periods before and after filling was measured by ecological indicators and by the presence and absence of some species/morphospecies. This is the first study, as far as we know, including taxonomic and ecological treatment to monitor the impact of a hydroelectric power plant on ant fauna.

**Data set 1.** 

Column label	Column description
eventID	An identifier for the set of information associated with an Event (something that occurs at a place and time).
eventDate	The date-time or interval during which an Event occurred. For occurrences, this is the date-time when the event was recorded.
eventTime	The time or interval during which an Event occurred.
habitat	A category or description of the habitat in which the Event occurred.
samplingProtocol	The name of, reference to, or description of the method or protocol used during an Event.
samplingEffort	The amount of effort expended during an Event.
eventRemarks	Comments or notes about the Event.
sampleSizeUnit	The unit of measurement of the size (time duration, length, area or volume) of a sample in a sampling event.
sampleSizeValue	A numeric value for a measurement of the size (time duration, length, area or volume) of a sample in a sampling event.
fieldNotes	The text of notes taken in the field about the Event.
continent	The name of the continent in which the Location occurs.
country	The name of the country or major administrative unit in which the Location occurs
countryCode	The standard code for the country in which the Location occurs.
stateProvince	The name of the next smaller administrative region than country (state, province, canton, department, region, etc.) in which the Location occurs.
county	The full, unabbreviated name of the next smaller administrative region than stateProvince (county, shire, department, etc.) in which the Location occurs.
locality	The specific description of the place.
locationRemarks	Comments or notes about the Location.
decimalLongitude	The geographic longitude (in decimal degrees, using the spatial reference system given in geodeticDatum) of the geographic center of a Location.
decimalLatitude	The geographic latitude (in decimal degrees, using the spatial reference system given in geodeticDatum) of the geographic center of a Location.
modified	The most recent date-time on which the resource was changed.
datasetName	The name identifying the data set from which the record was derived.
type	A list of nomenclatural types.
language	A language of the resource.
institutionID	An identifier for the institution having custody of the material referred to in the record.
institutionCode	The acronym in use by the institution having custody of the material referred to in the record.
rightsHolder	The organization owning the rights over the resource.

### Data set 2.

#### Data set name

Environmental monitoring of ants (Hymenoptera: Formicidae) in the influence areas of Santo Antônio Hydroelectric Power-Plant in Rondônia, Brazil.

#### Number of columns

33

#### Character set

Occurrence

#### 

**Data set 2.** 

Column label	Column description
ID	An identifier for the Identification (an identifier specific to the data set).
type	A list of nomenclatural types.
modified	The most recent date-time on which the resource was changed.
language	A language of the resource.
license	A legal document giving official permission to do something with the resource.
rightsHolder	The organization owning the material rights over the resource.
institutionID	An identifier for the institution having custody of the material referred to in the record.
institutionCode	The acronym in use by the institution having custody of the material referred to in the record.
datasetName	The name identifying the data set from which the record was derived.
basisOfRecord	The specific nature of the data record.
dynamicProperties	A list of additional measurements, facts, characteristics, or assertions about the record.
occurrenceID	An identifier for the Occurrence.
recordNumber	An identifier given to the Occurrence at the time it was recorded.
recordedBy	A list of names of people responsible for recording the original Occurrence.
organismQuantity	A number for the quantity of organisms.
organismQuantityType	The type of quantification system used for the quantity of organisms.
sex	The sex of the biological individual(s) represented in the Occurrence.
lifeStage	The age class or life stage of the biological individual(s) at the time the Occurrence was recorded.
reproductiveCondition	The reproductive condition of the biological individual(s) represented in the Occurrence.
preparations	A list of preparations and preservation methods for a specimen.
disposition	The current state of a specimen with respect to the collection identified in collectionCode or collectionID.
eventID	An identifier for the set of information associated with an Event (something that occurs at a place and time).
identifiedBy	A list of names of people who assigned the Taxon to the subject.
scientificName	An identifier for the nomenclatural details of a scientific name.
kingdom	The full scientific name of the kingdom in which the taxon is classified.
phylum	The full scientific name of the phylum or division in which the taxon is classified.
class	The full scientific name of the class in which the taxon is classified.
order	The full scientific name of the order in which the taxon is classified.
family	The full scientific name of the family in which the taxon is classified.
genus	The full scientific name of the genus in which the taxon is classified.
specificEpithet	The name of the first or species epithet of the scientificName.
taxonRank	The taxonomic rank of the most specific name in the scientificName.
vernacularName	A common or vernacular name.

## Additional information

Fernandes I (2017): Environmental monitoring of ants (Hymenoptera: Formicidae) in the influence areas of Santo Antônio Hydroelectric Power-Plant in Rondônia, Brazil. v1.7. Sistema de Informação sobre a Biodiversidade Brasileira - SiBBr. Dataset/Samplingevent. https://ipt.sibbr.gov.br/sibbr/resource?r=ant_monitoring_in_santo_antonio_hydroelectric_power_plant_rondonia&v=1.7

## Supplementary Material

Supplementary material 1 A total of 253 events of collection in the influence areas of Santo Antônio Hydroelectric Power-Plant. Data type: metadata (DwC-A) event
File: oo_182359.xlsx Itanna Oliveira Fernandes and Jorge Luiz Pereira de Souza

Supplementary material 2 A total of 9.165 occurrences in the influence areas of Santo Antônio Hydroelectric Power-Plant. Data type: metadata (DwC-A) occurences
File: oo_182361.xlsx Itanna Oliveira Fernandes and Jorge Luiz Pereira de Souza
